# A High‐Throughput Multi Well Plate–Based Approach for the Combined Expression, Export, and Assay of Recombinant Proteins

**DOI:** 10.1002/cpz1.70255

**Published:** 2025-11-06

**Authors:** Karen Baker, Daniel P. Mulvihill

**Affiliations:** ^1^ School of Biosciences University of Kent Canterbury United Kingdom

**Keywords:** biotechnology, drug screen, high‐throughput activity assay, protein engineering, protein production screen, recombinant proteins

## Abstract

High‐throughput screening (HTS) of proteins is used in a wide range of applications across the biology, biotechnology, and medicine disciplines. These include yield optimization, drug or biomarker discovery, and protein engineering, among others. Factors that need to be considered in designing high‐throughput protein expression and screening methods (be that for expression, activity, stability, or binding assays), include the required yield, reproducibility, solubility, stability, purity, and activity of the protein. Thus, larger culture volumes and time‐consuming manual protein extraction and purification steps are normally required to produce enough protein of appropriate purity. This limits the type of assay and number of protein variants that can be simultaneously tested in an experiment. Here, we describe a HTS protocol that allows the overnight expression, export, and assay of recombinant proteins from *Escherichia coli* cells in the same microplate well. The protocol uses a recently described Vesicle Nucleating peptide (VNp) technology that promotes high yield vesicular export of functional proteins from *E. coli* into the culture medium. The resulting protein is of sufficient purity and yield that it can be used directly in plate‐based enzymatic assays without additional purification. This simple single‐plate protocol allows itself to a wide range of high‐throughput research and development screening applications, ranging from streamlining protein production and identification of activity enhancing mutations, to ligand screening for basic research, biotechnological and drug discovery applications. © 2025 The Author(s). Current Protocols published by Wiley Periodicals LLC.

**Basic Protocol**: Expression, export, and isolation of vesicular‐packaged recombinant protein

**Support Protocol 1**: 96‐well plate cold‐shock transformation

**Support Protocol 2**: In‐plate affinity‐tag protein purification

**Support Protocol 3**: Example in‐plate enzymatic assay

## INTRODUCTION

High‐throughput screening (HTS) is a tool used routinely in drug discovery and discovery research to enhance protein production and rapidly evaluate millions of compounds, molecules, or proteins for activity against a biological target. Its efficiency and scalability make it an attractive method to optimize the molecular design and expression of functional proteins. High‐throughput protein expression/purification streamlines the rapid production and isolation of large numbers of proteins, thus reducing time and cost, accelerating both discovery research and drug discovery. Through application of simple, automated, and scalable workflows, the approach enables the parallel processing and testing of multiple protein constructs, conditions, and strains to optimize yield, solubility, functionality, and purity. It accelerates the identification of suitable targets for biochemical assays, structural analyses, and therapeutic development. This approach is particularly attractive in screening protein variants, producing enzymes with enzymatic activities optimized for industrial applications. The ability to quickly produce high‐quality proteins reduces time, cost, and bottlenecks in research pipelines, while at the same time enhancing reproducibility compared to traditional methods.

**Figure 1:**
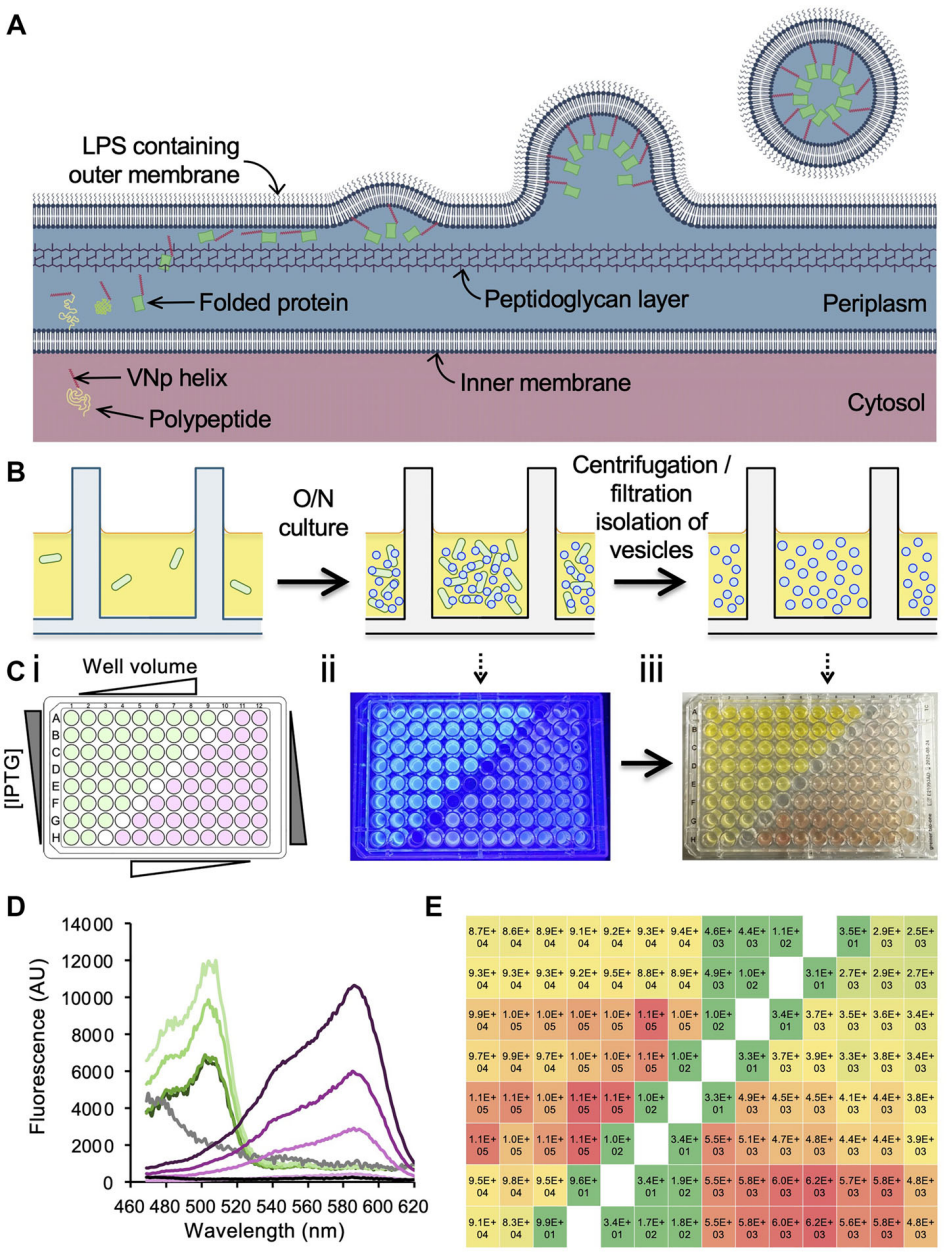
Multi‐well plate format recombinant protein expression and vesicular export screen. (**A**) Current model of mechanism that underlies the Vesicle Nucleating peptide (VNp) fusion‐dependent export of recombinant proteins into extracellular vesicles. VNp (red helix) tagged polypeptides pass from the cytosol (pink background) into the periplasmic space (blue), where they fold into mature proteins (green), pass through the peptidoglycan layer (purple) and interact with the outer membrane. Sufficient VNp–membrane interactions induce outward curvature of the membrane, and the formation of VNp fusion–filled membrane vesicles. (**B**) Schematic of multi‐well plate format workflow for recombinant expression and isolation of fusion protein filled extracellular vesicles. (**C**) 96‐well plate expression and vesicle export optimization screen of VNp‐mNeongreen (green) and VNp‐mCherry2 (pink). (i) Layout of VNp‐mNeongreen (green) and VNp‐mCherry2 (pink) expression construct containing *E. coli*, and test variables on the 96‐well plate. (ii) Blue light illuminated in plate cultures [organized as set out in (i) after overnight induction]. (iii) FP‐filled vesicle containing medium from (ii) after cell removal by plate‐well filtration. (**D**) Fluorescence scans of medium from (**C**iii). Pink lines highlight VNp‐mCherry2 abundance increases in proportion to culture volume [50 (light pink), 75, 100, and 150 (dark magenta) µl]. Green lines highlight variation in VNp‐mNeongreen abundance in medium from overnight cultures induced with 25 (light green), 50, 75, & 100 (dark green) µg/ml IPTG. (**E**) Heat map of mNeongreen (left) and mCherry2 (right) fluorescence in medium from overnight 96‐well plate format cultures, as described in (**B**).

We recently described the development of a Vesicle Nucleating peptide (VNp) technology (Eastwood et al., 2023) that provides an attractive alternative for the efficient production of recombinant proteins in *Escherichia coli*. The VNp tag facilitates the export of recombinant proteins into extracellular membrane‐bound vesicles (Fig. 1), creating a microenvironment that enhances protein solubility and stability, including those that are typically insoluble, contain disulfide bonds, or are toxic to the bacteria. The VNp tagging approach not only increases protein yield but exports the protein into vesicles in a partially purified form that allows for long‐term storage of active proteins.

The VNp technology requires fusion of a short amino‐terminal amphipathic alpha‐helix onto the protein of interest. This peptide interacts with the *E. coli* membrane, and at a critical concentration induces outward curvature of the lipid bilayer to form a vesicle that is released into the culture medium. This strategy allows the protein to be packaged and released from the cytosol, thus allowing further protein production by preventing localized cytosolic accumulation of protein that would otherwise impact the health of the cell. Due to basic differences in size and mass from the cells, the released vesicles can be easily isolated and transferred to a fresh plate by centrifugation. Once isolated, the vesicle can either be stored for >1 yr at 4°C in the sterile filtered medium, or membranes can be lysed by the addition of anionic or zwitterionic detergents and used directly in downstream assays. This protocol requires only a single sample transfer step between plates, during the vesicle isolation; however, if affinity purification of the protein is required, a single fresh plate transfer is necessary. While we routinely use multichannel pipettes when undertaking this high‐throughput multi‐well plate protocol, the method can be easily adapted for integration into robotic liquid handling systems. Whatever detailed strategy and assay you decide upon, the initial design of the VNp‐fusion construct is the most important factor for optimization of this protocol.

Monomeric globular proteins <85 kDa are likely to be exported into extracellular vesicles, but this is not a hard and fast rule. It is worthwhile testing expression and export of the desired VNp‐fusion using a flask culture as described in detail elsewhere (Streather et al., 2023), and then undertaking an expression and export optimization screen, as described here (Fig. 1). It is worth considering fusing your protein of interest with different amino‐terminal VNp tags (Eastwood et al., 2023) in combinations with mNeongreen, MBP, Sumo, or other solubilization tags and to determine the optimal basic construct for your application. Then, screen to determine the optimal combinations of medium, temperature, induction level, induction timing, and culture volume for production of the exported protein (Fig. 1).

While a wide range of *E. coli* protein expression HTSs have been described to date (Jia & Jeon, 2016; Saez & Vincentelli, 2014; Schreiber et al., 2017; Vincentelli et al., 2011), to our knowledge, this VNp‐based protocol is unique in that it represents the only HTS system allowing expression and vesicular export of a wide range of functional recombinant proteins from *E. coli* in a single step, without inducing cell lysis (Nettleship et al., 2019). The protocol described here is unique as it allows expression, export, and assay of a fusion protein in the same multi‐well format plate. Normally, this protocol would require separate larger volume steps for: (i) cell culture, protein expression, and cell isolation; (ii) cell disruption; (iii) protein extraction; (iii) lysate clarification; (iv) protein solubilization; and (v) purification, in order to isolate enough protein of sufficient purity for either enzymatic activity or binding assays (Black et al., 2017; Kemmer et al., 2023; Szmitkowska et al., 2020). The VNp system has the additional benefits of not only allowing expression of more challenging proteins but also expressing proteins at higher yields than standard methods (Eastwood et al., 2023). This VNp‐based protocol allows all the above stages to be completed in one step, as the protein is exported into the medium within vesicular packages in sufficient quantity and purity for a wide range of assays. This increased yield, combined with avoiding the need for cell disruption and subsequent protein purification and/or concentration steps, allows simple integration into a rapid multi‐well format HTS assay approach.

While several eukaryotic expression systems promote export of recombinant proteins (e.g., *Pichia pastoris* or mammalian cell culture) (Battle et al., 2006; Chapple & Dyson, 2021; Doyle et al., 2002; Fernández et al., 2019; Kalathur et al., 2016; Priola et al., 2016), these systems can be technically more challenging in regard to culture conditions, take longer times for growth and culture optimization, and have higher consumable costs. With this system typical optimized yields of exported VNp‐fusion range between 200 mg and 3 g per L of culture. With standard working volumes on multi‐well plates, these yields translate to typical yields of 0.2 to 3 mg (24‐well plates), 40 to 600 µg (96‐well plates), and 16 to 240 µg (384‐well plates) of exported, >80% purified protein. To our knowledge there are currently no equivalent HTS protocols available that are as quick, cheap, and easy to use or able to achieve equivalent reproducible yields and purities as that described here.

**Figure 2:**
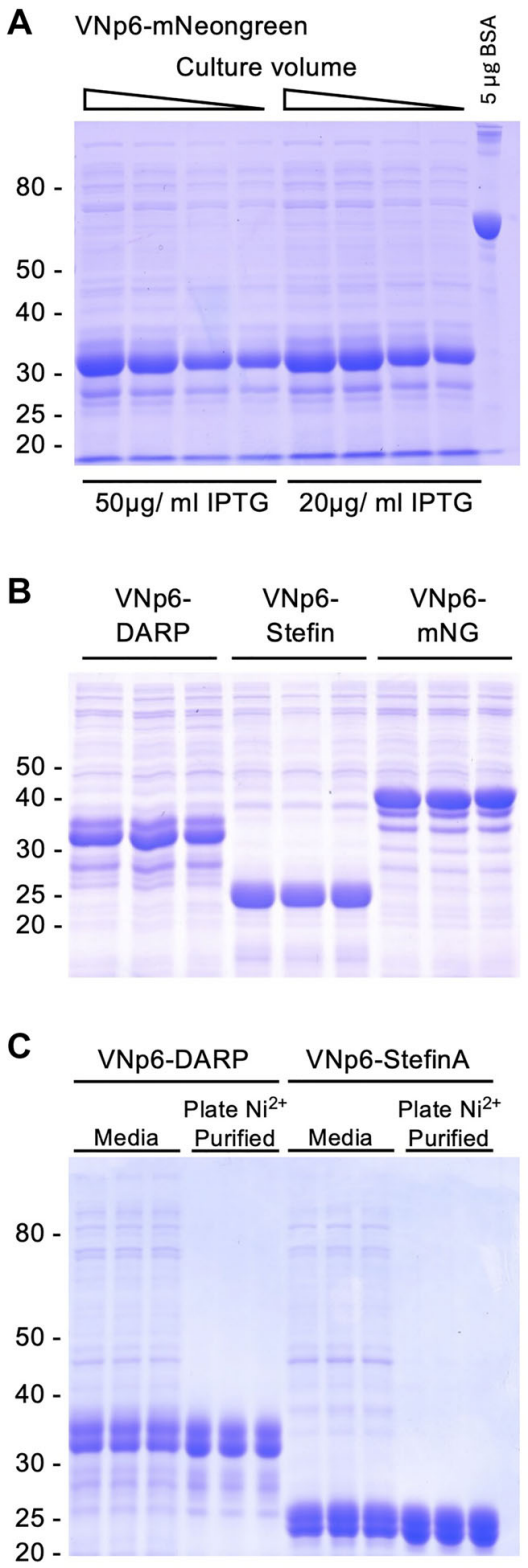
Examples of 96‐well plate format vesicular protein export and purification screen samples. Commassie stained SDS‐PAGE analysis of 10 µl media samples from different overnight 96‐well plate cultures of *E. coli* expressing (**A**) VNp6‐mNeongreen (samples from Fig. 1), (**B**) VNp6‐DARPin^off7^, VNp6‐Stefin‐A, or VNp6‐mNeongreen fusions, highlighting yield enhancement (**A**) and reproducibility (**B**). (**C**) SDS‐PAGE analysis of samples of media from 3 different overnight 96‐well plate cultures of *E. coli* expressing either VNp6‐DARPin^off7^ or VNp6‐Stefin‐A, as well as these carboxyl His_6_ tagged fusion‐proteins purified from the same media samples using the described in plate Ni^2+^‐NTA isolation (Support Protocol 2).

This HTS screen was developed for the rapid optimization of protein export conditions and subsequent ligand binding/inhibitor screens; however, it can be applied to a wide range of applications including yield optimization, expression libraries, drug or biomarker discovery, and protein engineering. A simple application of this protocol is to screen though a range of conditions to optimize expression and export of a protein of interest (Fig. 1). Only soluble, folded protein is exported into the vesicles (inclusions remain within the cytosol), the yields of folded protein can be determined using spectroscopy techniques. As shown here, the use of fluorescent protein fusions simplifies the analysis of protein expression and export (Fig. 1). However, expression, export, and purity of non‐fluorescent VNp‐fusions with the Protein‐of‐Interest (POI) can be followed by SDS‐PAGE analysis of the cell‐cleared medium (Fig. 2). Once conditions are optimized, expression and export of each VNp‐fusion is experimentally reproducible, making it perfect for use in screening applications that require equivalent amounts of protein (or proteins). However, if purified protein is required for downstream analysis or assays, the VNp‐fusion can be rapidly purified using the in‐plate affinity‐purification protocol included here (Fig. 2C) (Support Protocol 2).

**Figure 3:**
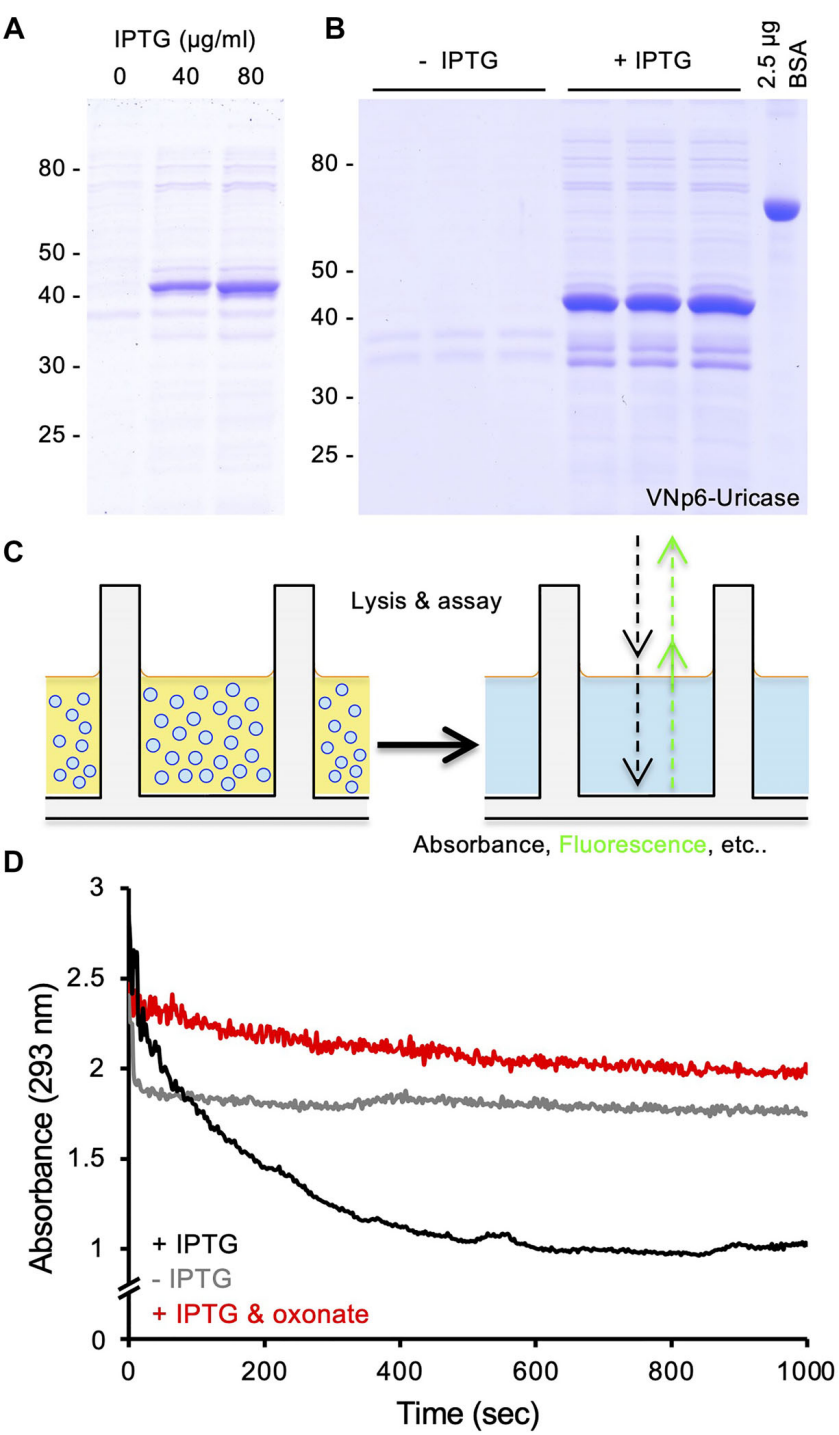
96‐well plate format screen of vesicular export, activity, and inhibition of active uricase. Commassie stained SDS‐PAGE analysis of filtered 10 µl media samples from overnight 96‐well plate cultures of *E. coli* containing a plasmid, expressing VNp6‐uricase fusion optimizing IPTG concentrations (**A**), or (**B**) of uninduced (–IPTG) and induced (+IPTG) samples for subsequent in‐plate uricase activity screen. (**C**) Schematic of vesicle cleavage and plate‐based assay. (**D**) 96‐well plate uricase assays (curves averaged from 4 experimental repeats) in wells with media samples lacking uricase release [from –IPTG sample in (**B**)] (grey line); with uricase released into the media [from +IPTG sample in (**B**)] (black line); or samples with uricase released into the media with addition of 20 µM uricase inhibitor, oxonate (red line).

The HTS protocol can be directly applied to follow enzymatic activity across samples within the plate. This is made possible due to the reproducibility, purity and yields (i.e., 40 to 600 µg of the VNp‐fusion protein typically obtained from a 100‐µl, 96‐well plate culture) (Fig. 2) (Eastwood et al., 2023). As an example, we describe a multi‐well format in vitro assay (Support Protocol 3) that allows researchers to measure the activity of in‐plate expressed and exported VNp‐uricase protein (Fig. 3). The measured enzymatic activities are reproducible between individual culture wells, a factor critical for high‐throughput protein engineering screens. Thus, by combining with a mutagenesis screen or mutant clone library, this protocol can be used to identify residues critical for protein function and allows researchers to rapidly engineer proteins with enhanced activity, resilience, and/or stability. Likewise, rapid biophysical and activity comparisons of HTS protein variants can be assessed.

The reproducibility of protein yields obtained using this protocol allows for its simple integration into ligand‐binding screen assays. Ligands, which can vary from small molecules to peptides, can be identified by either bringing about a change in absorbance/fluorescence upon binding to the protein of interest, or inhibiting/enhancing its activity, as demonstrated by inclusion of the uricase inhibitor, oxonate, in the in‐plate uricase assay (Fig. 3). The protocol can thus be used to efficiently screen small‐molecule drug libraries to identify protein effectors, and inversely, can also be used to express protein‐variants, protein domain (Kaushansky et al., 2010), or protein libraries (Cormier et al., 2012; Kohl et al., 2008; Rajagopala et al., 2010; Yang et al., 2011) to screen for specificity and efficacy of an effector ligand (Panavas et al., 2011).

The ability to be able to undertake high‐throughput in‐well ligand binding and activity assays of multiple sequence variants of the same protein allows researchers to rapidly identify key functional and structural residues within a protein important for ligand binding. Also, it provides a quick and cost‐effective method for producing protein banks/libraries. Use of the optional in‐plate purification step allows for the protocol's application in high‐throughput crystallography screens. Finally, the ability to transform different plasmids into a wide range of different *E. coli* strains using the cold‐shock steps described here, does away with the need to make banks of competent cells for each individual strain to be tested.

The protocol described here uses the VNp technology to rapidly express and isolate proteins from *E. coli* cells in a high‐throughput format. Basic Protocol describes a multi‐well plate method for the induction and isolation of VNp‐tagged recombinant proteins. Support Protocols 1 to 3 are included that can be used to support specific applications. Support Protocol 1 describes an in‐plate cold shock plasmid transformation that allows simultaneous introduction of multiple plasmids into cells, as well as simultaneous introduction into multiple *E. coli* strains. Support Protocol 2 describes a method for the in‐plate affinity purification of recombinant proteins if high purity is required for downstream applications. Support Protocol 3 describes an in‐plate enzymatic assay using the protein purified from Basic Protocol that can be easily adapted for a wide range of enzyme activity assays. These methods require minimal sample transfer between plates and can be easily adapted and applied to a plethora of high‐throughput research, development, and library screening applications to streamline protein production for biomedical research and industrial applications.

## Basic Protocol 1 EXPRESSION, EXPORT, AND ISOLATION OF VESICULAR‐PACKAGED RECOMBINANT PROTEIN

This protocol describes how to induce the expression and export of a VNp‐fusion protein from *E. coli* grown in multi‐well plate format cultures. It includes description of how to grow the cells, when to induce protein expression, and how to separate vesicles containing the exported protein from the cells.

### Materials

*E. coli* cells
*The VNp tags work with a range of strains (see* *Eastwood et al.*, 2023 *), but to date, the protocol works best with BL21 (DE3) Star for most proteins we have worked with*.
Bacterial plasmid(s) for expression of VNp‐fusions
*The plasmid should have non‐cell wall–targeting antibiotic selection marker (e.g., use tetracylcine, kanamycin, or chloramphenicol), and expression of the VNp‐fusion must be under the control of an inducible promoter (e.g., T7, T5, rhamnose, arabinose, etc.). A range of constructs generated by us are available from Addgene (* www.addgene.org/Dan_Mulvihill *)*.
VNp sequences:
  VNp2: MDVFMKGLSKAKEGVVAAAEKTKQGVAEAAGKTKEGVL
  VNp6: MDVFKKGFSIADEGVVGAVEKTDQGVTEAAEKTKEGVM
  VNp15: MDVFKKGFSIADEGVVGAVE
Terrific broth (TB) medium [12 g tryptone; 24 g yeast extract; 4 ml of 10% glycerol; 17 mM KH_2_PO_4_, 72 mM K_2_HPO_4_ (per L)]
Antibiotic (e.g., kanamycin, chloramphenicol, etc.)
Transcription induction reagent (e.g., IPTG, rhamnose, arabinose, etc.)
Triton X‐100 or Tween 20

V‐bottomed multi‐well plates (e.g., BrandTech cat. no. 781661 or Sarstedt, cat. no. 82.1583001)
*Use double orbital shaking during culture steps*.
Shaking UV and fluorescence plate reader (e.g., BMG Labtech CLARIOstar Plus) and/or shaking plate incubator (e.g., BMG THERMOstar)
Gas‐permeable plate sealing film (e.g., BrandTech, cat. no. 701365)
Centrifuge with plate adapters (e.g., Eppendorf 5920R, cat. nos. 5948000060 and 5895125000)
Multi‐well filter plates (e.g., Whatman Unifilter Microplate, cat. no. 7700‐3308)
Flat‐bottomed multi‐well assay plates (e.g., Greiner, cat. no. 655180)
Black UV transmission multi‐well assay plates (e.g., BrandTech cat. no. 781615)
*If using absorbance spectroscopy for protein yield or if undertaking enzymatic assays*.

*NOTE*: See Current Protocols (2006) for commonly used reagents.

1 Use a standard heat‐shock method, as described in any standard laboratory manual, e.g., Sambrook and Russell (2001), to introduce plasmid into required *E. coli* strain.
  If introducing multiple plasmids or using multiple E. coli strains, we recommend using Support Protocol 1 instead.
2 The following morning, either pick single colonies (if following on from step 1) or aliquot 5 µl from overnight culture wells (if using Support Protocol 1) and transfer into wells of a V‐bottomed 96‐well plate containing up to 200 µl TB + antibiotic (Fig. 1).
  Optimize the culture volume for your construct and downstream screen/application. (Fig. 1B‐D).
3 Grow on heated shaking plate incubator (or preferably shaking plate reader with double orbital shaking) at 37°C, 200 rpm until cultures reach a late log phase density (typically <2 hr).
  Optional step: Once cells reach the desired density, change culture temperature at this point, if required (and using VNp6 tag) for optimized protein folding.
4 Induce protein expression by addition of transcription inducer molecule.
  Typically use up to 50 µg/ml IPTG with T5 and T7 promoters.
5 Seal plate surface with gas‐permeable film to minimize evaporation of medium.
6 Culture overnight at 37°C with 200 rpm shaking (double orbital shaking if available).
7 After at least 12 hr, separate cells from medium using one of the following methods.

##### Option A

7a Centrifuge 5 min at 2250 × *g*, 4°C.
7b Transfer supernatants onto required assay plate.

###### Option B

7a Transfer onto 0.45‐µm filter plate mounted onto required assay plate.
7b Centrifuge 2 min at 2250 × *g*, 4°C. Vesicles and medium will pass through to the fresh plate.
8 Place plate containing medium (and exported vesicles) at 4°C.
  Recombinant protein and vesicles can remain stable in sterile medium for at least a year.
9 Lyse vesicle membranes by addition of non‐ionic detergent (e.g., 0.1 % Triton X‐100 or 0.1 % Tween 20).

#### Outcome

Each well now contains independently produced partially purified VNp‐fusion protein. The abundance, quality, and purity of each sample can now be assessed using standard techniques (e.g., SDS‐PAGE or spectroscopy) and used in a downstream assay (Fig. 1C‐D; Fig. 2).

## Support Protocol 1 96‐WELL PLATE COLD‐SHOCK TRANSFORMATION

This protocol describes an in‐plate cold‐shock transformation method for the introduction of a library of different constructs into *E. coli* cells, as well as the simultaneous introduction of plasmid(s) into different *E. coli* strains. This multi‐well plate format protocol allows a range of constructs to be simultaneously tested (e.g., use for comparing different promoters, VNp tags, fusion protein mutants/variants), as well as the introduction of plasmid(s) into different *E. coli* strains, and thus negates the requirement for competent cell preparation.

### Materials

*E. coli* cells (see Basic Protocol)
Plasmid DNA (see Basic Protocol)
Lysogeny/Luria‐Bertani broth (LB) medium [10 g tryptone; 10 g NaCl; 5 g yeast extract (per L)]
100 mM CaCl_2_ + 10% glycerol
Dry ice
Industrial methylated spirits (IMS)
LB agar plate with antibiotic (e.g., kanamycin, chloramphenicol, etc.)

V‐bottomed multi‐well plates (e.g., BrandTech cat. no. 781661 or Sarstedt, cat. no. 82.1583001)
Shaking UV and fluorescence plate reader (e.g., BMG Labtech CLARIOstar Plus) and/or shaking plate incubator (e.g., BMG THERMOstar)
Centrifuge with plate adapters (e.g., Eppendorf 5920R, cat. nos. 5948000060 and 5895125000)
Ice bucket and ice

*NOTE*: See Current Protocols (2006) for commonly used reagents.

1 Inoculate wells in a V‐bottomed plate, each containing 100 µl LB medium, with *E. coli* cells at 37°C using a shaking plate incubator/plate reader set at 200 rpm agitation (use double orbital setting if available).
2 Once most cultures have reached an OD_600_ of 0.6 to 0.8, harvest the cells by centrifugation for 5 min at 2250 × *g*, 4 °C, and aspirate off the medium.
3 Resuspend in 50 µl of ice‐cold 100 mM CaCl_2_ + 10% glycerol. Leave on ice for 10 min.
4 Add 1 µl of plasmid DNA from a 0.01 to 0.1 µg/µl stock to each well.
5 Mix by agitation then place on ice for 10 min.
6 Place onto a bath of dry ice and IMS for 90 s
  Put layer of dry ice at bottom of ice bucket and pour over with industrial methylated spirits (so base of plate is resting in liquid).
7 Immediately place at 37°C to thaw (typically 5 min).
8 Add 100 µl LB and incubate for 1 hr at 37°C to recover.
9 Take ≥5 µl volumes from each well and grow transformants overnight using one of the following 2 methods.

##### Option A

9a Plate out onto a matrix grid on an LB agar plate supplemented with appropriate antibiotic.
9b Incubate at 37°C overnight.
  Ensure LB agar plate has dried sufficiently to efficiently absorb culture aliquots.
9c Once colonies are visible in the morning, store plate at 4°C until ready to use.

###### Option B

9a Add to fresh V‐bottom multi‐well plate with wells containing LB supplemented with appropriate antibiotic.
9b Culture overnight at 37°C with shaking.
10 Once colonies are visible in the morning, store the plate at 4°C until ready to use.
  Plate can be stored up to 1 week at 4°C.

## Support Protocol 2 IN‐PLATE AFFINITY‐TAG PROTEIN PURIFICATION

If >80% protein purity is required for your downstream application/assay, use this in plate affinity purification protocol to purify your protein of interest.

### Materials

Recombinant protein plates (see Basic Protocol 1)
Affinity resin (e.g., Ni^2+^‐NTA) (Thermo Scientific, cat. no. 88221)
Wash buffer (20 mM Tris, 500 mM NaCl, 25 mM imidazole, pH 8.0)
Elution buffer (20 mM Tris, 500 mM NaCl, 300 mM imidazole, pH 8.0)

Shaking UV and fluorescence plate reader (e.g., BMG Labtech CLARIOstar Plus) and/or shaking plate incubator (e.g., BMG THERMOstar)
Centrifuge with plate adapters (e.g., Eppendorf 5920R, cat. nos. 5948000060 and 5895125000)
Flat‐bottomed multi‐well assay plates (e.g., Greiner, cat. no. 655180)

*NOTE*: See Current Protocols (2006) for commonly used reagents.

1 Add 10 µl affinity resin (e.g., Ni^2+^‐NTA) to each medium‐containing well (V‐bottomed plates are optimal for centrifugation).
2 Mix on shaker at 4°C for 30 min.
3 Centrifuge 2 min at 500 × *g*, 4°C.
4 Wash in 100 µl wash buffer. Repeat steps 3 and 4 twice more.
5 Isolate protein from resin with elution buffer.
6 Spin 2 min at 500 × *g*, 4°C. Transfer the buffer, containing purified protein, to a fresh flat‐bottomed multi‐well plate for assay (Fig. 2C).

## Support Protocol 3 EXAMPLE IN‐PLATE ENZYMATIC ASSAY

Vesicles used in this example were isolated, using the method described above, from BL21 DE3 star *E. coli* cells containing the pRSFDUET‐VNp6‐uricase‐His_6_ expression plasmid (Eastwood et al., 2023) (Addgene plasmid cat. no. 182401).

### Materials

Uric acid (Fisher Scientific, cat. no. 11410973)
100 mM Tris, pH 8.5 (Current Protocols, 2006)
Exported protein sample (see Basic Protocol 1, step 9)

Black UV transmission multi‐well assay plates (e.g., BrandTech cat. no. 781615)
Shaking UV and fluorescence plate reader (e.g., BMG Labtech CLARIOstar Plus) and/or shaking plate incubator (e.g., BMG THERMOstar)

1 Add 50 µl of 2 mM uric acid (dissolved in 100 mM Tris, pH 8.5) to UV transmission flat‐bottomed 96‐well plate.
2 Using a plate reader, measure absorbance at 293 nm every 1 s at 25°C for 1 min.
3 Add 50 µl of exported protein sample from Basic Protocol, step 9.
  Add inhibitors, test inhibitors, etc. to samples at this step, if required.
4 Immediately continue monitoring absorbance at 293 nm every 1 s at 25°C until end of reaction in all wells. (Fig. 3)

## COMMENTARY

### Background Information

To improve the throughput and enhance efficiency of expression and export of the VNp‐fusion protein, we developed this multi well plate–based protocol to allow a range of conditions to be tested simultaneously (e.g., volume, cell type, transcription induction levels, medium, VNp variant, etc.), to rapidly optimize growth, expression and export of the VNp‐fusion. This screening method has been used successfully for construct design and optimization of conditions to express, produce, and package a range of functional proteins (e.g., IgG fusions, EPO, hGH, and monoclonal antibodies) (Baker et al., 2025; Eastwood et al., 2023). The simplicity of the VNp expression and vesicular export system allows itself to rapid fusion protein isolation, with only a single centrifugation (or filtration) step required to isolate the fusion‐protein filled vesicles from the *E. coli* cells. As the subsequent VNp‐fusion protein within the vesicle fraction is relatively pure (typically >85% of total vesicular protein), and of high yield (typically ranges from 0.2 to 3 g/L), it is well suited for integration into many HTS assay needs. This multi‐well plate method can easily be extended to facilitate in‐plate enzymatic assay of the exported proteins, for protein engineering, and ligand binding/inhibitor assays.

### Critical Parameters

#### Construct design and E. coli strain selection

We have generated and tested a large number of Vesicle Nucleating peptides (VNps), and for most applications would recommend using either the VNp2, VNp6 or VNp15 sequences (see Star Methods section in Eastwood et al., 2023), as these routinely result in highest yields for most proteins tested to date. When fused to the amino terminus of a protein, these short (38 or 20 residues) peptide tags are sufficient to promote association with the membrane and subsequent encapsulation into a vesicle. While it may be attractive to fuse the VNp to the carboxyl terminus of some proteins, it is less likely to result in vesicular export using this strategy, and even when it does protein yields are significantly lower.

Protein and assay specific requirements for the design of the final fusion (e.g., affinity purification, solubility, fluorescence tags, labeling residues, spacers, etc.) should be considered. While there is a lot of flexibility in the choice of backbone for the expression construct plasmid, there are two important constraints. The first is the selection marker. Using an antibiotic marker that affects the bacterial cell wall (e.g., ampicillin) dramatically reduces the viability of cells when expressing VNp‐fusion. Therefore, we routinely use the non‐cell wall antibiotics markers kanamycin, chloramphenicol, or tetracycline for construct selection, as these do not measurably affect VNp‐fusion export. Second, expression of the VNp‐fusion must be under the control of a regulatable promoter as expression levels need to be repressed during transformation and be regulatable during expression to optimize vesicular export. To date we have used rhamnose, arabinose, and IPTG (both T5 and T7) inducible promoters, which together allow a range of options for repression and induction levels, as well as a wide choice of *E. coli* expression strains. The VNp system works best in B and K12 *E. coli* strains and routinely use BL21 (DE3) Star cells for most VNp‐fusion protein expression applications. However, we have successfully used other *E. coli* strains when necessitated by the protein and/or assay.

#### Transformation, culture, and induction

In this protocol, if required, we apply bulk cold‐shock transformation to introduce a library of different constructs into different *E. coli* cells within the multi‐well plate format. This does away with the need to make competent cells and allows a range of constructs to be simultaneously tested (e.g., use for comparing different promoters, VNp tags, fusion protein mutants/variants, strains, or different combinations of each) in a range of different *E. coli* strains. The transformation efficiency is more than sufficient to introduce a plasmid into cells in this format and bypasses the requirement to create competent cells for each strain used.

This protocol can be used to assay one plasmid (e.g., to identify optimal conditions) or multiple plasmids or multiple strains. If the former, in order to maximize consistency between cultures, a single starter culture should be used to inoculate each well. If the latter, individual starter cultures should be set up in each well. Most standard *E. coli* media can be used for this protocol, and while TB is routinely used to maximize both cell growth and protein expression, glycerol concentration should be kept to a minimum (Eastwood et al., 2023) to maximize vesicle formation. If minimal medium is required for downstream labeling during the assay (e.g., N^15^ labeling for NMR applications), cells should be initially cultured in rich medium and transferred to minimal medium at induction.

Expression should be induced at late log phase, at a typical OD_600_ of 0.8 to 1.0. Maximal induction of VNp‐fusion proteins, using standard promoters, leads to rapid cell lysis, and therefore recombinant protein transcription levels should be moderated. For example, IPTG should be used at a reduced final concentration of 20 µg/ml for most VNp‐fusions expressed from genes under the control of the T7 promoter. It is worth noting that optimal protein production and optimal export conditions do not always coincide with each other, and therefore a balance needs to be struck to obtain maximal export of recombinant protein for the subsequent assay. For example, while vesicle formation is most efficient when cells are cultured at 37°C (lipid membranes are more dynamic), some proteins, in order to fold correctly, require expression at lower temperatures. In these situations, we tend to recommend using the VNp6 tag, which promotes membrane reorganization across a wider range of temperatures.

One important internal control that should be considered when using this protocol, is to include control wells containing equivalent cells expressing a non‐VNp labelled variant of protein to confirm VNp‐dependent export and assay specificity. However, cell growth should be monitored during optimization of the protocol to highlight optimal growth conditions and identify cell death brought about by overexpression of the VNp‐fusion.

#### Vesicle isolation, protein release, and purification or assay

Harvesting vesicles requires separation from the cell culture and transfer to a fresh multi‐well plate. This can be done easily by either centrifugation or filtration centrifugation. Cells can be pelleted with a 5 min spin, and the vesicle containing medium can be carefully transferred to a fresh multi‐well plate, appropriate for the assay (e.g., black wells, UV transparent, etc.). Alternatively, the cultures are transferred to an appropriate multi‐well filter that retains cells but allows vesicles to pass through. Whichever method is used, the plate containing samples of protein filled vesicles can either be stored long term at 4°C at this stage or used directly. To release protein into the medium, vesicles are lysed in the plate by adding non‐ionic detergent (e.g., Triton, Tween, etc.). The protein can be assayed directly or purified using a simple in plate affinity purification protocol. The protein can then be used directly in any fluorescence or absorbance‐based assay as required. We include the description of an enzymatic assay here to illustrate the functionality of vesicle packaged proteins and demonstrate how this protocol produces sufficient protein in multi‐well plates to measure rate changes in signal with a HTS format. It is worth noting that the vesicle lysis step is only required if the assay substrate is unable to pass across a lipid membrane.

### Troubleshooting

See Table 1 for a troubleshooting guide for expression, export, and assay of recombinant proteins.

**Table 1:** Troubleshooting Guide for Expression, Export, and Assay of Recombinant Proteins

Problem	Possible cause	Solution
No protein expression	Plasmid selection antibiotic choice	Do not use an antibiotic that affects the cell wall
	Cell density at induction	Induce fusion protein expression when the cells are reaching late log phase
	VNp‐fusions induce cell lysis if expressed at high levels	Use moderate levels of transcriptional inducer (e.g., 20‐50 µg/ml IPTG)
No vesicular export of protein from the cell	VNp construct design	Ensure VNp tag is at amino terminus of the final fusion; add a linker peptide [e.g., (GGSG)_2_] between VNp and protein of interest
	Protein is insoluble	Add solubility tag (e.g., mNeongreen or MBP) between VNp and protein of interest
	Insufficient/too much VNp‐fusion produced to allow outward protein export	Optimize protein induction levels
	Protein is incorporated into intracellular vesicles	Optimize protein induction levels and culture conditions; possibly due to size/biophysical properties of your protein
	Culture volume not optimal	Use method to screen for optimal culture volume and induction levels (Fig. 1)
	Culture temperature	VNp vesicle induction is optimal at 37°C; if lower temperatures are required, use the VNp6 tag
	Medium	Vesicle induction is inhibited in high [glycerol]; use LB and/or TB recipes described here
	*E. coli* strain not optimal	Use a BL21 or K12 *E. coli* strain [BL21 (DE3) star is optimal]
Proteins are not binding to affinity column/ligands	Vesicles still intact	Use higher concentration of the detergent
Enzyme inactive in assay	Enzyme has no/low activity in culture medium	Use Support Protocol 2 to purify the protein and resuspend in optimal activity buffer
	Vesicles still intact	Use higher concentration of the detergent

### Understanding Results

The aim of this protocol is to express recombinant protein in a multi‐well plate to optimize protein production for downstream protein engineering, and/or ligand binding screens. Results from example expression and assay screens are presented here to illustrate expected outcomes. As a simple example, we include a HTS assay for the optimization of mNeongreen and mCherry2 fluorescent protein VNp fusions (Fig. 1B). In the example assay, vesicular export is examined in *E. coli* strains grown in increasing culture volumes and IPTG concentrations (Fig. 1Ci). After an overnight culture the wells fluoresce (Fig. 1Cii), and once the cells have been removed, the medium can be seen to be colored by the fluorescent protein (Fig. 1Ciii). To quantify yields, absorbance spectra were obtained for the samples (Fig. 1C) using a plate reader, and the subsequent relative yields determined (Fig. 1D). Interestingly, in this example, smaller volumes result in an increase in mNeongreen yield, while the opposite is the case for the VNp‐mCherry2 fusion, with larger culture volumes resulting in improved yields (Fig. 1C), demonstrating different proteins require different growth conditions. Adding a fluorescent tag to simplify the screening can be applied to almost any protein of interest, and as the case for mNeongreen, can also increase the protein solubility.

Using quantification by absorbance, the results are consistent with analysis by Coomassie stained SDS‐PAGE analysis of the samples (Fig. 2A). The yields can be estimated from the stained gels by running known quantities of a protein standard, e.g., BSA (Fig. 2A and 3B). This method can also be applied to compare samples from a HTS where the protein lacks fluorescence (Fig. 2B). As can be seen from these gels, most of the protein present in the medium (≥80%) is the VNp‐fusion, and this level of purity is suitable for many downstream applications (e.g., Fig. 3). However, if high purity protein is required, the in‐plate affinity purification described in Support Protocol 2, can be used to rapidly purify your VNp‐fusion protein into a fresh plate (Fig. 2C).

The final example presented here illustrates the optimization of a VNp‐uricase fusion protein production, and subsequent analysis in an in plate enzymatic assay (Fig. 3). The results show optimization of protein expression levels (Fig. 3A), yields and reproducibility (Fig. 3B). These samples were then used directly in a uricase assay, where one monitors the breakdown of uric acid by following changes in absorbance at 293 nm (Fig. 3D). In this example enzyme assays are shown for the medium from cells grown in the absence (grey line) or presence of IPTG (black line), or the presence of both IPTG and oxonate, a uricase inhibitor (red line) (Fig. 3D).

### Time Considerations

A standard bacterial transformation takes ∼2 hr, including a 1 hr recovery step essential for bactericidal antibiotic selection. The cold‐shock multi‐well plate transformation method described in Support Protocol 1 requires ∼ 3 hr. This represents a significant time saving, when time for competent cell preparation is considered. For both methods, the transformations are left overnight to grow and can be used directly the following morning for the Basic Protocol, or they can be stored at 4°C for up to 1 week. For Basic Protocol itself, the setup takes ∼5 min and once growing, the cells are ready for induction typically within 2 to 3 hr (OD_600_ can be followed in real time if a plate reader incubator is used). After adding the transcription induction reagent to each well, the wells are sealed with gas permeable membrane and then left overnight to grow. The following morning it takes ∼10 min to separate cells from the vesicle containing medium (by centrifugation or filtration). If the protein needs to be further purified, the multi‐well affinity purification described in Support Protocol 2 takes another 10 to 20 min. Finally, if all the required reagents are prepared beforehand, the uricase activity assay (Support Protocol 3) typically takes 25 to 30 min. While there are many points throughout the whole procedure where the protocol can be paused and the samples stored at 4°C until required, using the freshest possible sample is always advisable.

### Author Contributions

**Karen Baker**: Conceptualization; investigation; writing–review and editing. **Daniel Mulvihill**: Conceptualization; funding acquisition; supervision; writing–original draft; writing–review and editing.

### Conflict of Interest

The authors declare no conflict of interest.

## Data Availability

Primary data can be made available upon request to the corresponding authors.

## References

[cpz170255-bib-0001] Baker, K. , Eastwood, T. A. , Garcia, E. , Lennon, C. , & Mulvihill, D. P. (2025). Simple recombinant monoclonal antibody production from *E. coli* . Open Biology, 15. 10.1098/rsob.240229 PMC11835484 39965660

[cpz170255-bib-0002] Battle, T. , Antonsson, B. , Feger, G. , & Besson, D. (2006). A high‐throughput mammalian protein expression, purification, aliquoting and storage pipeline to assemble a library of the human secretome. Combinatorial Chemistry & High Throughput Screening, 9, 639–649. 10.2174/138620706778700143 17100570

[cpz170255-bib-0003] Black, C. , Barker, J. J. , Hitchman, R. B. , Kwong, H. S. , Festenstein, S. , & Acton, T. B. (2017). High‐throughput production of proteins in *E. coli* for structural studies. Methods in Molecular Biology, 1586, 359–371. 10.1007/978-1-4939-6887-9_24 28470618

[cpz170255-bib-0004] Chapple, S. D. , & Dyson, M. R. (2021). High‐throughput expression screening in mammalian suspension cells. Methods in Molecular Biology, 2199, 117–125. 10.1007/978-1-0716-0892-0_7 33125647

[cpz170255-bib-0005] Cormier, C. , Park, J. , Fiacco, M. , Steel, J. , Kramer, J. , & LaBaer, J. (2012). Protein structure initiative: Biology‐materials repository: Developing a public resource for structural biology plasmids. The FASEB Journal, 26, 985.1–985.1. 10.1096/fasebj.26.1_supplement.985.1

[cpz170255-bib-0006] Current Protocols . (1994). Extraction and Precipitation of DNA. Current Protocols in Human Genetics, 00, A.3C.1–A.3C.4. 10.1002/0471142905.hga03cs00 18428221

[cpz170255-bib-0007] Doyle, S. A. , Murphy, M. , Primus, J. , Richardson, P. , & Hawkins, T. (2002). High‐throughput protein expression and purification for proteomics research. The Scientific World Journal, 2, 146–147. 10.1100/tsw.2002.71 29973846 PMC6009278

[cpz170255-bib-0008] Eastwood, T. A. , Baker, K. , Streather, B. R. , Allen, N. , Wang, L. , Botchway, S. W. , Brown, I. R. , Hiscock, J. R. , Lennon, C. , & Mulvihill, D. P. (2023). High‐yield vesicle‐packaged recombinant protein production from *E. coli* . Cell Report Methods, 3, 100396. 10.1016/j.crmeth.2023.100396 PMC10014274 36936078

[cpz170255-bib-0009] Fernández, F. J. , Gómez, S. , & Vega, M. C. (2019). High‐Throughput Protein Production in Yeast. Methods in Molecular Biology, 2025, 69–91. 10.1007/978-1-4939-9624-7_4 31267449

[cpz170255-bib-0010] Jia, B. , & Jeon, C. O. (2016). High‐throughput recombinant protein expression in *Escherichia coli*: Current status and future perspectives. Open Biology, 6. 10.1098/rsob.160196 PMC5008019 27581654

[cpz170255-bib-0011] Kalathur, R. C. , Panganiban, M. , & Bruni, R. (2016). High‐throughput baculovirus expression system for membrane protein production. Methods in Molecular Biology, 1432, 187–202. 10.1007/978-1-4939-3637-3_12 27485337

[cpz170255-bib-0012] Kaushansky, A. , Allen, J. E. , Gordus, A. , Stiffler, M. A. , Karp, E. S. , Chang, B. H. , & MacBeath, G. (2010). Quantifying protein‐protein interactions in high throughput using protein domain microarrays. Nature Protocols, 5, 773–790. 10.1038/nprot.2010.36 20360771 PMC3085283

[cpz170255-bib-0013] Kemmer, A. , Cai, L. , Cruz Bournazou, M. N. , & Neubauer, P. (2023). High‐throughput expression of inclusion bodies on an automated platform. Methods in Molecular Biology, 2617, 31–47. 10.1007/978-1-0716-2930-7_3 36656515

[cpz170255-bib-0014] Kohl, T. , Schmidt, C. , Wiemann, S. , Poustka, A. , & Korf, U. (2008). Automated production of recombinant human proteins as resource for proteome research. Proteome Science, 6, 40–10. 10.1186/1477-5956-6-4 PMC2266735 18226205

[cpz170255-bib-0015] Nettleship, J. E. , Rada, H. , & Owens, R. J. (2019). Overview of a high‐throughput pipeline for streamlining the production of recombinant proteins. Methods in Molecular Biology, 2025, 33–49. 10.1007/978-1-4939-9624-7_2 31267447

[cpz170255-bib-0016] Panavas, T. , Lu, J. , Liu, X. , Winkis, A. ‐ M. , Powers, G. , Naso, M. F. , & Amegadzie, B. (2011). Generation and evaluation of mammalian secreted and membrane protein expression libraries for high‐throughput target discovery. Protein Expression and Purification, 79, 7–15. 10.1016/j.pep.2011.05.011 21640830

[cpz170255-bib-0017] Priola, J. J. , Calzadilla, N. , Baumann, M. , Borth, N. , Tate, C. G. , & Betenbaugh, M. J. (2016). High‐throughput screening and selection of mammalian cells for enhanced protein production. Biotechnology Journal, 11, 853–865. 10.1002/biot.201500579 27276699

[cpz170255-bib-0018] Rajagopala, S. V. , Yamamoto, N. , Zweifel, A. E. , Nakamichi, T. , Huang, H. ‐ K. , Mendez‐Rios, J. D. , Franca‐Koh, J. , Boorgula, M. P. , Fujita, K. , Suzuki, K. ‐ I. , Hu, J. C. , Wanner, B. L. , Mori, H. , & Uetz, P. (2010). The *Escherichia coli* K‐12 ORFeome: A resource for comparative molecular microbiology. BMC Genomics, 11, 133–8. 10.1186/1471-2164-11-470 20701780 PMC3091666

[cpz170255-bib-0019] Saez, N. J. , & Vincentelli, R. (2014). High‐throughput expression screening and purification of recombinant proteins in *E. coli* . Methods in Molecular Biology, 1091, 33–53. 10.1007/978-1-62703-691-7_3 24203323

[cpz170255-bib-0020] Sambrook, J. , & Russell, D. W. (2001). Molecular cloning: A laboratory manual, the third edition. Cold Spring Harbor Laboratory Press.

[cpz170255-bib-0021] Schreiber, C. , Müller, H. , Birrenbach, O. , Klein, M. , Heerd, D. , Weidner, T. , Salzig, D. , & Czermak, P. (2017). A high‐throughput expression screening platform to optimize the production of antimicrobial peptides. Microbial Cell Factories, 16, 29–13. 10.1186/s12934-017-0637-5 28193216 PMC5307881

[cpz170255-bib-0022] Streather, B. R. , Baker, K. , Eastwood, T. A. , & Mulvihill, D. P. (2023). Optimized Production and Analysis of Recombinant Protein‐Filled Vesicles from E. coli. Journal of visualized experiments : JoVE, (196). 10.3791/65442 37458456

[cpz170255-bib-0023] Szmitkowska, A. , Pekárová, B. , & Hejátko, J. (2020). A high‐throughput strategy for recombinant protein expression and solubility screen in *Escherichia coli*: A case of sensor histidine kinase. Methods in Molecular Biology, 2077, 19–36. 10.1007/978-1-4939-9884-5_2 31707649

[cpz170255-bib-0024] Vincentelli, R. , Cimino, A. , Geerlof, A. , Kubo, A. , Satou, Y. , & Cambillau, C. (2011). High‐throughput protein expression screening and purification in *Escherichia coli* . Methods, 55, 65–72. 10.1016/j.ymeth.2011.08.010 21925268

[cpz170255-bib-0025] Yang, X. , Boehm, J. S. , Yang, X. , Salehi‐Ashtiani, K. , Hao, T. , Shen, Y. , Lubonja, R. , Thomas, S. R. , Alkan, O. , Bhimdi, T. , Green, T. M. , Johannessen, C. M. , Silver, S. J. , Nguyen, C. , Murray, R. R. , Hieronymus, H. , Balcha, D. , Fan, C. , Lin, C. , … Root, D. E. (2011). A public genome‐scale lentiviral expression library of human ORFs. Nature methods, 8(8), 659–661. 10.1038/nmeth.1638 21706014 PMC3234135

